# Stromal Vascular Fraction for Osteoarthritis of the Knee Regenerative Engineering

**DOI:** 10.1007/s40883-021-00226-x

**Published:** 2021-08-11

**Authors:** Chinedu C. Ude, Shiv Shah, Kenneth S. Ogueri, Lakshmi S. Nair, Cato T. Laurencin

**Affiliations:** 1 Connecticut Convergence Institute for Translation in Regenerative Engineering, Farmington, CT, USA; 2 Raymond and Beverly Sackler Center for Biomedical, Biological, Physical and Engineering Sciences, University of Connecticut Health, Farmington, CT, USA; 3 Department of Orthopaedic Surgery, University of Connecticut Health, Farmington, CT, USA; 4 Department of Chemical and Biomolecular Engineering, University of Connecticut, Storrs, CT, USA; 5 Department of Biomedical Engineering, University of Connecticut, Storrs, CT, USA; 6 Department of Materials Science and Engineering, University of Connecticut, Storrs, CT, USA; 7 Institute of Materials Science, University of Connecticut, Storrs, CT, USA; 8 Department of Craniofacial Sciences, School of Dental Medicine, University of Connecticut Health, Farmington, CT, USA

**Keywords:** Osteoarthritis, Knee regeneration, Adipose tissue, Stromal vascular fraction

## Abstract

**Purpose:**

The knee joint is prone to osteoarthritis (OA) due to its anatomical position, and several reports have implicated the imbalance between catabolic and anabolic processes within the joint as the main culprit, thus leading to investigations towards attenuation of these inflammatory signals for OA treatment. In this review, we have explored clinical evidence supporting the use of stromal vascular fraction (SVF), known for its anti-inflammatory characteristics for the treatment of OA.

**Methods:**

Searches were made on PubMed, PMC, and Google Scholar with the keywords “adipose fraction knee regeneration, and stromal vascular fraction knee regeneration, and limiting searches within 2017–2020.

**Results:**

Frequently found interventions include cultured adipose-derived stem cells (ADSCs), SVF, and the micronized/microfragmented adipose tissue-stromal vascular fraction (MAT-SVF). Clinical data reported that joints treated with SVF provided a better quality of life to patients. Currently, MAT-SVF obtained and administered at the point of care is approved by the Food and Drug Administration (FDA), but more studies including manufacturing validation, safety, and proof of pharmacological activity are needed for SVF. The mechanism of action of MAT-SVF is also not fully understood. However, the current hypothesis indicates a direct adherence and integration with the degenerative host tissue, and/or trophic effects resulting from the secretome of constituent cells.

**Conclusion:**

Our review of the literature on stromal vascular fraction and related therapy use has found evidence of efficacy in results. More research and clinical patient follow-up are needed to determine the proper place of these therapies in the treatment of osteoarthritis of the knee.

**Lay Summary:**

Reports have implicated the increased inflammatory proteins within the joints as the main cause of osteoarthritis (OA). This has attracted interest towards addressing these inflammatory proteins as a way of treatment for OA. The concentrated cell-packed portion of the adipose product stromal vascular fraction (SVF) from liposuction or other methods possesses anti-inflammatory effects and has been acclaimed to heal OA. Thus, we searched for clinical evidence supporting their use, for OA treatment through examining the literature. Data from various hospitals support that joints treated with SVF provided a better quality of life to patients. Currently, there is at least one version of these products that are obtained and given back to patients during a single clinic visit, approved by the FDA.

## Introduction

Osteoarthritis (OA) has become the most prevalent joint disease in the elderly population [1–4] as well as in young active individuals such as athletes and servicemen [5]. Osteoarthritis most frequently affects the cells and tissues in the knee joints, owing to its anatomical position and function, causing impairments in mobility and the quality of life [3, 6]. Treatment strategies include targeting all the affected regions of the joints to reduce joint pain and restore normal functional capacities [6].

Physical therapy is one early intervention for OA that helps to strengthen the muscles around the joints attenuating pain and atrophy [7]. Data suggest that patients can also benefit from knee braces and shoe orthotics [4, 8]. Nutriceuticals, such as glucosamine and chondroitin sulfates, have also been beneficial in maintaining cartilage health and improving mobility [3]. Patients can benefit from oral non-steroidal anti-inflammatory drugs, as well as corticosteroids and hyaluronic acid (HA) [9]. Fluoroscopic and ultrasound-guided neural blockade can be applied to certain patients for pudendal and sympathetic nerve blockades [10]. However, when patients are not getting relief of their symptoms and their joint approaches end-stage degeneration, arthroplasty is recommended [8, 11]. Arthroplasty has been beneficial, however, but it can be accompanied by serious complications [6, 11].

Early techniques to promote joint regeneration and cartilage self-healing have been focused on abrasion arthroplasty, microfracture, and subchondral drilling [4, 6]. It was believed that the released blood beneath the lesion can contain native/pluripotent cells, linking bone marrow stem cells’ migration to elicit regeneration [12].

Considering that OA is partly caused by the imbalance between the catabolic and anabolic processes within the joints, several reports implicated inflammatory mechanisms to correlate with the cause of structural degeneration [13, 14]. This has led to exploring anti-inflammatory activities of cell/cellular products for OA treatment [1, 8, 15]. The identification of resident native cells dates back to the 1860s when Cohnheim demonstrated the existence of nonhematopoietic, plastic adherent, and fibroblastic-like cells from bone marrow for wound healing [16]. In the 1960s, Friedenstein confirmed that the cells form colonies from single cells and coined the term colony-forming unit fibroblastic (CFU-Fs) [14]. Based on their ability to differentiate into three mesodermal lineages, Caplan, in the 1990s, named these CFU-Fs, mesenchymal stem cells (MSCs) [17, 17].

MSCs are critical for tissue repair and in maintaining the body’s homeostasis by having the ability to differentiate into different connective tissue lineages [13, 19]. MSCs have been found in numerous tissues including bone marrow, umbilical cord blood, skin, muscles, periosteum, synovial membrane, and adipose tissues [20–23]. Recently, native cells in adipose tissues have attracted great attention due to their abundance in the body, ease of accessibility, and regenerative capabilities [16, 24]. Adipose-derived stem cells (ADSCs), stromal vascular fractions (SVFs), and micronized/microfragmented adipose tissue-stromal vascular fractions (MAT-SVFs) are among the most frequently reported cell interventions [25]. These cellular products are believed to evoke their regenerative effect through the secretion of bioactive molecules that act in a paracrine fashion to prime and sustain angiogenic, anti-fibrotic, anti-apoptotic, and immuno-modulatory responses in the target tissues [3, 10, 21, 26].

This review focuses on recent clinical evidence of adipose-based SVF and MAT-SVF, their mechanism of action, limitations, regulatory challenges, and future directions of these cells on knee OA treatment.

## Adipose Tissues for Regenerative Engineering

Adipose tissues can be harvested from various fat depots in the body, namely, the abdomen, waist, thigh, hips, buttocks, and the infrapatellar fat pad of the knee joint [25, 27]. These are loose connective tissues composed of native uncommitted cells (adipose stem cells), preadipocytes (Adipose cells precursor), and mature adipocytes [24, 25]. In addition, adipose tissues contain stromal vascular-related cells, including fibroblasts, vascular endothelial cells, pericytes, and a variety of immune-related cells, such as macrophages and monocytes [21, 28]. Their main role is to store energy in the form of lipids, cushion delicate organs, and insulates the body [29, 30]. They can be classified according to their color and location in the body. The white adipose tissue, also known as visceral fat, organ fat, or intra-abdominal fat, which stores energy is located within the peritoneal cavity, packed in between the internal organs and torso [25, 25]. The brown adipose tissues, which generate body heat, are located underneath the skin, known as subcutaneous fat, and interspaced in the skeletal muscles, known as intra-muscular fats [25, 32]. Adipose tissue also constitutes part of major endocrine organs and is hormonally active (active hormones include, leptin, estrogen, and resistin) [3, 14].

### Adipose-Derived Therapies

In recent years, there has been an increased call by several stakeholders to modify and clarify the different regenerative cocktails from adipose tissues and lipoaspirate [24]. In line with this, reports from various studies evaluating adipose-based therapies for knee joint degenerations can be categorized into ADSCs, SVF, and MAT [12, 29]. These different therapies will be discussed below.

### Adipose-Derived Stem Cells

Adipose-derived stem cells are native/undifferentiated adipose tissue cells [16], uncommitted to any lineage or phenotype, and retain similarity to cells first identified in the bone marrow [22]. The earliest adipose-derived stem cells were isolated from human liposuction aspirates called processed lipoaspirate (PLA) cells [33]. The adipose-derived cells shared similar characteristics with MSCs [24], exhibiting plastic adherence, fibroblast-like morphology, self-renewal, and capacity for multipotent differentiation [22]. It was then suggested that human PLA cells were perhaps a clonal variant of the MSC, located within the adipose compartment; hence, these multipotent adipose-derived cells could be a suitable alternative for invasively obtained bone marrow stem cells (BMSCs) [20]. Many groups have designated different names to these cells [16, 20, 21]; however, the International Federation for Adipose Therapeutics and Science (IFATS) adopted the term adipose-derived stem cells (ADSCs) to identify them [6, 20]. ADSC utility depends on their in vitro expansion, which comes with its challenges, including possible loss of stemness and transformation risks.

### Stromal Vascular Fraction

**Fig. 1:**
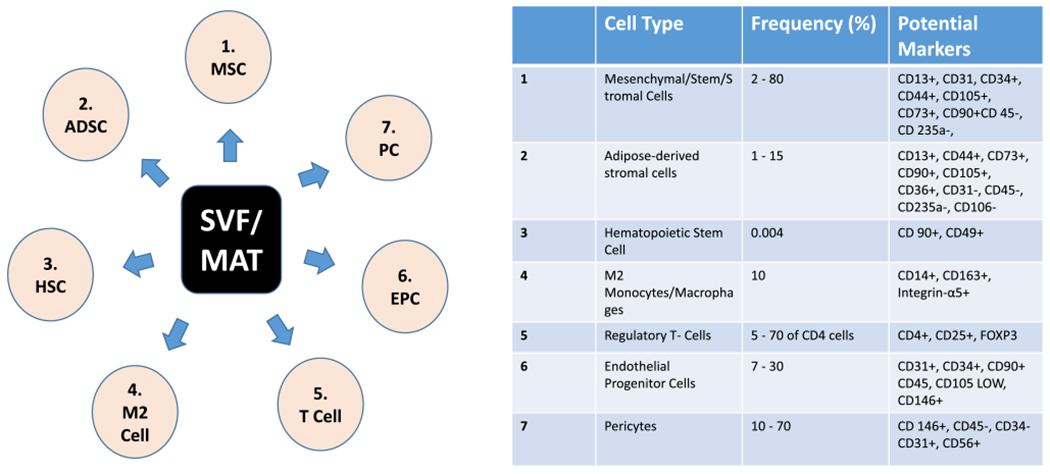
A Different cells that make up SVF and MAT-SVF. **B** Cells, frequency of occurrence, and associated markers of SVF and MAT-SVF

Stromal vascular fraction (SVF) is a collection of non-expanded, heterogeneous cells and stromal tissues obtained through liposuction [11, 31] that are derived through enzymatic digestion of lipoaspirate [16]. The SVF cell population includes hematopoietic cells, endothelial progenitors, endothelial cells, adipose-derived stem cells, adipose progenitors, fibroblasts, pericytes, immune cells, macrophages, leukocyte subtypes, lymphatic cells, smooth muscle cells, and other uncharacterized cells (Fig. 1) [10]. The percentage of ADSCs in SVF ranges between < 1 and > 15%; however, it may vary substantially depending on the patient’s age, health, and harvesting method [25]. Unlike ADSCs therapy that needs in vitro expansion, SVF does not need expansion [30, 32]. Other advantages of SVF include the heterogeneous cellular composition, which aids better therapeutic outcomes [15, 20]. In addition, the presence of pericytes, in SVF, serves vital roles in regeneration by differentiating into activated MSCs in response to injury and inflammation [10, 32]. However, the isolation method for SVF, including collagenase digestion, erythrocyte lysis, and centrifugation, can negatively affect the original composition.

#### Micronised Adipose Tissue-Stromal Vascular Fraction

In recent years, the need to address FDAs’ regulations prohibiting collagenase processed SVF products, coupled with inventions of intra-operative procedures that deliver autologous cells back into the patient without further processing, led to the development of MAT-SVF [12, 14, 30]. MAT-SVFs are obtained from micromincing, fragmentation, and homogenization of lipoaspirate adipose contents, without enzymatic digestion and cell expansion [16, 25]. The isolation procedure involves mechanical agitation, which breaks down the component tissue matrix and releases the stem cells. The distinguishing factors of the MAT-SVF to SVF include the microvascular structure of extracellular matrix (ECM) that remains intact and the native adipose ECM structure that provides a niche for other cellular subsets of bioactive, enabling more biologic functions, cell migration and modulation, cell signaling, interaction, and differentiation [7, 25]. Comparative studies have shown that enzymatic disruption of adipose tissues resulted in a completely different composition compared to mechanical disruption of adipose tissue, which mechanically condenses the tissue by selective removal of adipocytes without damaging key components[2, 29]. However, the cellular yield from MAT methods is lower than those from the enzymatic SVF methods, as cells from adipose tissue are still bound by collagen networks. It has been reported that the number of nucleated cells in MAT-SVF and SVF methods may range between 1 × 10^5^ and 1.5 × 10^6^ nucleated cells/mL respectively of lipoaspirate content [14, 33]

## Isolation and Characterization of Adipose-Derived Therapies

### Isolation of SVF and MAT-SVF

A popular technique for isolating SVF from adipose tissues starts from liposuction [34]. The harvested adipose is washed to remove lipoaspirate fluids. The fatty portion undergoes digestion with collagenase type 1. The digests are separated into upper mature adipocytes with liquid fat fraction and lower cellular fraction. The cellular fractions are spun into a reddish SVF pellet [2, 24, 25]. Red blood cells are removed from the pellets and the remaining total nucleated cells would be the SVF [13, 24]. Then, for MAT-SVF, instead of enzymatic digestion, the upper mature adipocytes would be separated from the lower cellular fraction through centrifugation [25, 29]. The cellular fraction would further be minced or mechanically agitated to microfragments to release stromal cells [14].

**Fig. 2:**
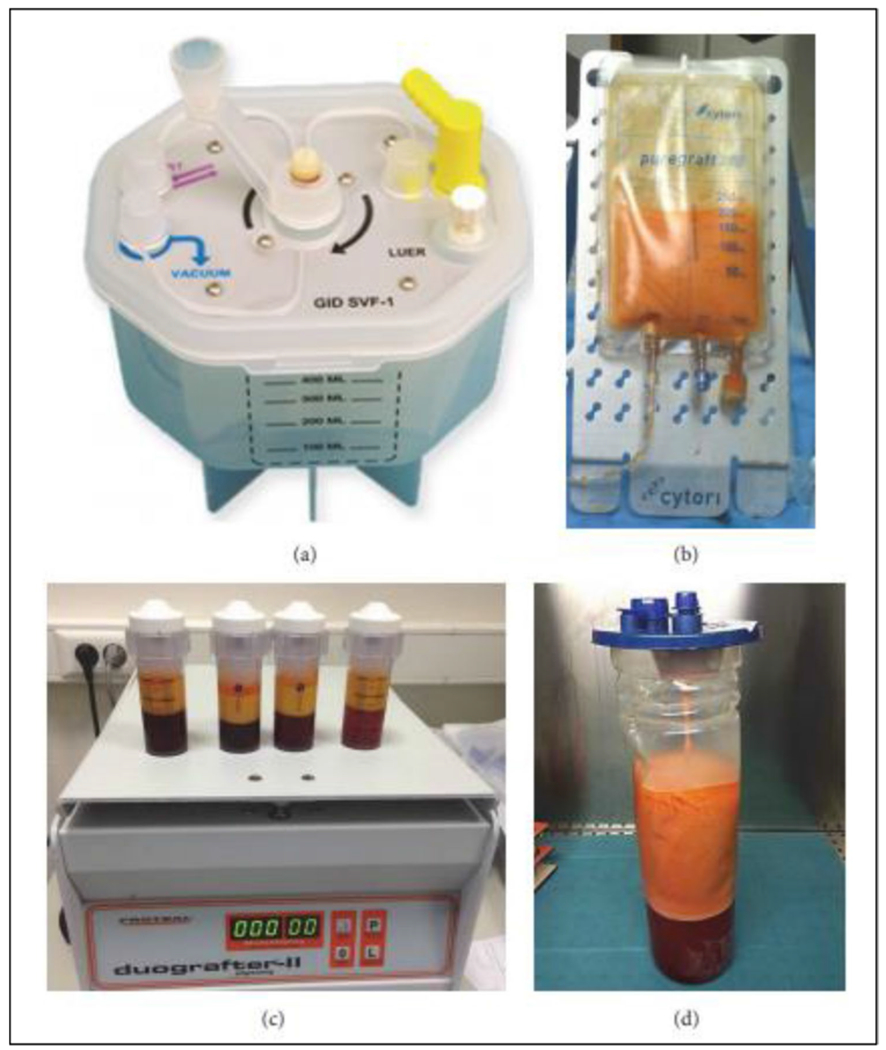
Devices used for SVF and SVF-MAT isolation. **a** GID-SVF1. **b** Puregraft 250. **c** Stem.pras. **d** Reference method. Adapted from Jonathan Rodriguez et al., 2017 [35]

New processing kits/technologies have been developed by different cell therapy companies to obtain ready-to-use, autologous SVF and MAT products (Fig. 2) (Table 1) [14, 16, 24, 29]. Simple non-enzymatic isolation methods utilizing, physical forces, mechanical segregation strategies in gravity separation, centrifugation, and mechanical emulsification have been favored to circumvent the safety issues posed by enzymatic protocols [35]. Some of them are optimized to provide greater cell viability, as well as a lower percentage of contaminants [15, 16]. Furthermore, in addition to the reduced production costs, protocol duration, and regulatory concerns, the non-enzymatic isolation methods have improved precise separation by the use of automated or semi-automated closed systems, to standardize operator-dependent variations, thereby making isolation and therapeutic application more approachable in clinics [8, 30].

### Identification and Morphology of SVF and MAT-SVF

The concentrated fractions of both SVF and MAT-SVF have a reddish appearance; however, if SVF is treated with erythrocyte lysis buffer to remove red blood cells, the isolated fraction would appear less reddish. MAT on the other hand contains more matrix in the pelleted concentrate, being that they were only microfragmented; hence, microvasculatures, reduced adipose clusters/adipose niche, and released exosomes remain intact [3, 28]. Despite mixed cell populations, they still contain a population that possesses the basic characteristics of MSCs [10]. Phenotypically, the International Society for Cellular Therapy (ISCT) and the IFATS have also established minimum criteria for the identification of SVF and MAT in relation to ADSCs (Fig. 1B) [20]. Generally, surface markers of ADSCs are similar to those of BMSCs with more than 90% overlap [33]. They have a positive expression to CD13, CD44, CD73, CD90, and CD105, and negative expression to CD31, CD45, and CD235a. However, ADSCs’ positivity for CD36 and negativity for CD106 could be used to distinguish both cells [14, 33]. SVF could be characterized with the minimal criteria for MSCs, and in addition to CD45−, CD235a−, CD31−, and CD34 + [10, 14, 33]. After isolation, CD34 is found in 80% of SVF and MAT; furthermore, the positivity to CD34 increases to 95% 2 days after the initial culture. However, the expression is lost during multiple expansions, hence the difference between CD34 + and CD34 − cell population could be used to distinguish SVF, MAT, and cultured ADSCs [20, 33].

## Clinical Data for Knee Regeneration Using SVF

### The Stromal Vascular Fraction Treatment for Osteochondral Knee Defect: Case Report

A 36-year-old patient who sustained knee injuries skydiving was admitted with complaints of pronounced pain in the right knee that got worse after physical exercise for up to 8 months, despite conservative treatments and physical therapy. Magnetic resonance imaging (MRI) indicated a traumatic osteochondral lesion of the medial femoral condyle, an underlying significant bone bruise, with a high risk of osteonecrosis and posttraumatic osteoarthritis. Clinical examination revealed significant atrophy of the right thigh muscles and a range of motion between 0 and 100° at the right knee. Pre-treatment arthroscopic evaluation showed a grade 3 local chondromalacia in the medial femoral condyle, and a mini-arthrotomy revealed a cartilage lesion of about 5 cm^2^. Microfracture was performed, in addition to 1.5 million autologous SVF cells injected with fibrin sealant Tissucol Kit, using a Duploject system. Results revealed a gradual reduction in pain, 6 months post-treatment. The range of motion in the right knee joint improved to 140°. The patient returned to the previous level of physical activity, with mild periodic pain. Pre-treatment International Knee Documentation Committee (IKDC) score was 23; post-treatment, IKDC was 56 at 3 months and 96 at 24 months [31].

### Arthroscopic Findings of Cartilage and Meniscus Repairs after Injection of SVF in Knee Osteoarthritis: Case Report

A 54-year-old male occupational therapist had knee pain in both legs for over 7 months. Conservative treatments failed, leading to a gradual withdrawal from active sports. Physical examination showed mild swelling with a positive McMurray test and tenderness on the medial joint space in both knees. X-ray and MRI showed Kellgren Lawrence (KL) grade II–III medial cartilage loss and the degeneration of medial meniscus. Pre-treatment diagnostic arthroscopy showed a 2.5 × 1.5 cm cartilage defect at the right femoral condyle with a grade III lesion according to the International Cartilage Repair Association (ICRS) classification. The left medial femoral condyle had a 3 × 1.5 cm cartilage tear of ICRS grade III. Twelve million viable SVF cells were injected into the intra-articular space of both knees after aspiration of 0.5 mL synovial fluid. Four weeks post-treatment, the patient reported knee pain reduction with an improvement of Knee Injury and Osteoarthritis Outcome Scores (KOOS) without any rehabilitation. Six months later, the patient returned to sports, and post-treatment arthroscopy showed regenerated cartilages on the medial and lateral condyles in both knees [8].

### Arthroscopic Findings of Femoral and Tibial Condyle Repairs After Injection of SVF in Knee Osteoarthritis: Case Report

A 63-year-old with right knee pain for more than a year had continuous deterioration, and limitations in active sport, despite all conservative treatments including hyaluronic acid injections. Physical examination revealed mild swelling and tenderness on the medial joint space in the right knee. X-ray and MRI indicated osteophyte formation and degenerative changes on the medial meniscus. Pre-treatment arthroscopy showed partial-thickness cartilage tear of ICRS grade II, with 2 × 1.5 cm fibrillation in the medial femoral condyle and 1.5 × 1.5 cm in the tibial medial condyle. Five million viable adipose-derived regenerative cells were injected into the intra-articular space after synovial fluid aspiration. Pain and functional recovery improved 1 month after treatment without rehabilitation. Six months post-procedure, the patient returned to active sports, and post-treatment arthroscopy showed complete regeneration on the medial femoral condyle, and repair of the posterior horn of the medial meniscus [8].

### Level Three Cohort Clinical Trial of Adipose-Derived Stem Cells and SVF for the Treatment of Knee Osteoarthritis

In a clinical study, cultured adipose-derived stem cells and non-cultured SVF for the treatment of knee osteoarthritis were studied in 80 patients. Forty-two patients (59 knees) received 1.28 × 10^7^ ADSCs, and 38 patients (69 knees) received a 5 mL SVF injection. All patients had KL grade (2–4) knee osteoarthritis and had failed medical management. Follow-up evaluations were completed by the visual analog scale (VAS) pain score and KOOS at baseline, and up to 6 months post-injections. The results indicated that SVFs had a higher frequency of knee effusion (8%), compared to ADSCs (2%), and harvest site complications, SVF (34%), and ADSC (5%). In the ADSC treated group, pain VAS and KOOS domains improved by 3 months, and pain VAS decreased by 55% compared to the SVF group (44%). The proportion of Outcome Measures-Osteoarthritis Research Society (OMERACT-OARSI) responders in the ADSC group was slightly higher (61%), compared to SVF (55%). It was concluded that both groups (ADSCs and SVFs) resulted in clinical improvement in patients with knee OA; however, ADSCs outperformed SVF in the early reduction of symptoms and pain with few complications [19].

### Non-Randomized, Phase I/II Trial to Evaluate the Improvement in Knee Pain, Function, and Cartilage Restoration

In a single-center, non-randomized, phase I/II trial to evaluate the improvement in knee pain, and function, as well as cartilage restoration, 33 patients (> 38 years) with KL (III) knee osteoarthritis were evaluated. Patients with commodities and intra-articular injections within 3 months were excluded. About 100 mL abdominal fats were harvested from each patient, with Triport Harvester Cannula, and 6 mL of SVF containing 90–120 million cells were isolated. Microfracture was done by arthroscopy, and the knee joints were drained before the injection of the SVF. Follow-up results indicated a decreased trend in visual analog scale (VAS) score and Western Ontario and McMaster Universities Osteoarthritis (WOMAC) index in the SVF-treated group up to 24 months, as compared with the placebo group. A significant increase in the Lysholm and a decrease in the Outerbridge Score (OS) scores were observed in the SVF treated group. The WOMAC scores were reduced in the KL3 groups, indicating more improvement in the KL3 group. Furthermore, the bone marrow edema (BME) was significantly decreased in the SVF group within 24 months [4].

### Double-Blinded Randomized Study to Evaluate the Efficacy of Intra-Articular Injection of SVF for Knee Osteoarthritis Treatment

Thirty-nine patients between 40 and 75 years old with symptomatic knee OA were recruited into three groups: high-dose SVF (3.0 × 10^7^ cells), low-dose SVF (1.5 × 10^7^ cells), and placebo group (zero cells). SVF was injected intra-articularly. WOMAC and MRI evaluations were obtained pre-operatively. At 6 months post-injection, WOMAC scores for each group were 83.9% for the high-dose group, 51.5% for the low-dose group, and 25.0% for the placebo group. The high- and low-dose groups had statistically significant changes compared with the placebo group. Furthermore, the median percentage changes in WOMAC from baseline to 1-year post-treatment for the high-dose, low-dose, and placebo groups were 89.5%, 68.2%, and 0%, respectively, reflecting that the high- and low-dose groups had greater changes at 12th months compared with the placebo; however, the improvements were dose-dependent [3].

### Double-Blind Randomized Self-Controlled Trial Comparing the Efficacy of Autologous Adipose-Derived SVF Versus HA

Sixteen patients with bilateral symptomatic knee osteoarthritis (KL grade II–III) were randomized into two groups. Each patient received a single dose of SVF injection in one knee joint (test knee, *n* = 16) and HA in the contralateral knee (control knee, *n* = 16). Patients were seen for follow-up at 1, 3, 6, and 12-months post-injection. The SVF-treated knees showed significantly improved mean VAS, WOMAC, and range of motion (ROM) scores at 12 months, compared to baseline; the HA group became worse. Whole organ magnetic resonance score (WORMS) and magnetic resonance observation of cartilage repair tissue (MOCART) measurements revealed significant improvements in cartilage repair in SVF-treated knees compared with HA knees, suggesting that autologous adipose-derived SVF can improve function and repair cartilage defects of knee osteoarthritis [9].

### Long-Term Multi Centric Case Study on the Efficacy of SVF for OA in Very Elderly Population Whose Conservative Managements Have Failed

A total of 29 patients, between 80 and 94 years old (males 31.1%, and females 68.9%), were recruited to evaluate the efficacy of SVF. Initial evaluation utilizing the K-L scale, clinical examination, X-ray, and/or MRI revealed that 10.3% of the patients were at grade 2, 48.3% with grade 3, and 41.4% with grade 4 degenerations. SVF was intra-articularly or peri-articularly administered into the knee or other affected joints. Complex score evaluations (pain, number of analgesics/NSAIDs per week, limping at a walk, joint stiffness, and extent of joint movement) by the modified Knee/Hip Osteoarthritis Outcome Score (KOOS/HOOS) depicted pain as well as the total amount of NSAIDs were significantly decreased from the first-month post-SVF therapy and significantly decreased 36 months post-SVF therapy. Similar results were obtained on limping at a walk, extent of joint movement, and stiffness. Data suggests that SVF represents an important tool in the regeneration of joints in elderly patients [11].

## Clinical Evidence of Knee Regenerations With SVF-MAT

### Retrospective Observational Study on the Treatment Efficacy of Autologous MAT for Diffuse Degenerative Knee Osteoarthritis

Thirty patients with different degrees of diffused degenerative chondral lesions were treated in the study. About 24 patients had an associated anterior cruciate ligament/lateral cruciate ligament (ACL/LCL) reconstruction, high tibial osteotomy, and meniscectomy, while 6 had undergone arthroscopy. Clinical outcomes were determined using KOOS, International Knee Documentation Committee (IKDC)-subjective, Tegner Lysholm Knee, and VAS pain scales. Improvement of 10 points was selected as clinically significant. Results revealed no relevant complications and a total of 20 points median improvement in IKDC and KOOS scores. Furthermore, improvements were also recorded in VAS pain and Tegner Lysholm Knee, 21 and 31 points, respectively, thereby supporting the safety and feasibility of autologous MAT in treating diffuse degenerative chondral lesions [7].

In a 3-year follow-up, all 30 patients shared the presence of diverse degrees of chondral lesions and had received intra-articular injections of MAT. The results showed no adverse events (harvesting site, or inflammations of treated joints) for 29 patients. On average, 22 patients had no other treatments in the 3-year follow-up. Furthermore, the patients improved in their Tegner Lysholm Knee, VAS, IKDC-subjective, and total KOOS evaluations, 41%, 55%, and 64%, respectively. More than 50% of the patients improved more than 20 points on the VAS pain scale and about 55% of the patients improved more than 30 points on the VAS pain scale. However, one patient died, and seven (23%) received additional treatments [17].

### Efficacy of Combining HSC and SVF for Treatment of Orthopedic Degenerative Diseases

SVF was injected into 58 patients through direct intra-articular injection or indirect intravenous injection to the bloodstream. Evaluation and potential benefits of treatment were done through standard questionnaires. Post-treatment follow-up on 44 of the 58 patients revealed that gender, age, clinical condition, certain SVF doses, and route of injection did not play a role in the clinical outcome. Furthermore, combining SVF and expanded-HSCs did not show increased efficacy compared to SVF injection alone. However, it was suggested that a ratio of 2:1 ADSCs to HSC-progenitors was very potent in knee regeneration [33].

### Clinical Investigation to Evaluate the Effect of Articular Injections of Autologous Concentrated Adipose Fraction for Knee OA Treatment

Adipose tissues were harvested from subabdominal fat, and MAT was processed to collect ADSCs before injection to the knees of 20 patients. The outcomes were monitored through VAS and WOMAC scores 3–18 months post-injections. Results depicted that all patients had improvement in pain and functional recovery. Newly formed tissues were visualized by immunohistochemistry (IHC) staining and scanning electron microscope (SEM), while joint biopsies showed new tissue formations. Overall, these data indicated that concentrated adipose fraction injections are safe for knee OA and could stimulate tissue regeneration [15].

### Prospective, Non-Randomized, Open-Label Clinical Investigation Analyzing Functional Efficacy of Intra-Articular Injection of Autologous MAT, and the Classification of Cell Types Contributing to Treatment

Twenty patients, between 40 and 80 years old, with late-stage primary knee OA, KL grade III (*n* = 4), and IV (*n* = 16), were enrolled. Adipose tissue was obtained and MAT was processed. MAT was injected intra-articularly to the index knees, and follow-up evaluations were done 12-months post-treatment. Results revealed that 17 patients (85%) showed a significant improvement in their KOOS and WOMAC scores. KOOS score improved 176% when compared with baseline, WOMAC decreased from 40 to 45%, and VAS scores decreased from 54 to 82%, depicting a positive effect of MAT on late-stage knee OA. Endothelial progenitor cells, pericytes, and supra-adventitial adipose stromal cells were the most abundant cell phenotypes [2].

### Randomized Controlled Single Blinded Investigation to Evaluate Clinical Outcomes in Patients Affected with Symptomatic Focal Chondral Lesions of the Knee Treated with MAT Plus Microfractures

Forty patients (40 knees) with femoral condyle OA grades III–IV, Outerbridge classification, were randomized into two groups. Both groups had initial arthroscopic microfractures for their focal chondral knee defects. Furthermore, patients in the treatment group had about 50 mL of abdominal fat harvested, and 10 mL of MAT obtained through the Lipogems® techniques were injected into their knee under arthroscopy. Follow-up was done at 1, 3, 6, and 12 months post-interventions, and the primary endpoint was WOMAC score at 12 months. At 3 months, patients in both groups improved from baseline in all variables, while at 6 and 12 months, patients in the MAT group scored better outcomes; in particular, better WOMAC scores (17.7 ± 11.1 vs. 25.5 ± 12.7; *p* = 0.03), thus achieving the primary end point of the study. It was concluded that injection of MAT plus microfractures is more effective in treating focal chondral lesions than microfractures alone [29].

### Prospective Study Evaluating the Safety and Effectiveness of Autologous SVF Therapy for Knee OA

This large-scale prospective study, comprising of 2586 participants, focused on evaluating the safety and effectiveness of autologous SVF therapy for knee OA. SVF was injected into the affected knee or knees. In some cases, platelet-rich plasma (PRP) was added to the intra-articular deployment. Due to the high number of patients in the study, evaluations were based on patients’ responses recorded as a simple visual acuity pain score of 0 (no pain) to 10 (worse pain they could tolerate) for (1) at rest, (2) standing, (3) walking, and (4) running. Data were collected from all participating affiliates of the cell surgical network through a HIPAA compliant online database (TrackVia.com). The results indicated an overall 82% improvement by autologous SVF application. This was evidenced even in chronic knee arthritis, with statistically significant functional recoveries from pain and mobility within 1 to 2 years. There were no gender differences in outcomes; all BMI levels showed improvements. Furthermore, there was no difference between SVF alone and with the addition of PRP [10].

### Evaluation of Safety and Clinical Efficacy of Freshly Isolated Autologous SVFs in Patients with Grade 2–4 (KL) Degenerative Osteoarthritis

In a large case-controlled, multi-centric non-randomized trial involving 1128 patients, the investigation was carried out by a team of International Consortium for Cell Therapy and Immunotherapy (ICCTI) in the USA, the Czech Republic, Slovakia, and Lithuania to evaluate the safety and clinical efficacy of freshly isolated autologous SVFs in patients with grade 2–4 (KL) degenerative osteoarthritis. SVF was administered intra-articularly or peri-articularly to the knee and/or other joints. Post-treatment follow-up was up to 54 months and the Modified KOOS/HOOS Clinical Score was used for assessments 12 months post-treatment. Results revealed no serious side effects and most patients’ condition gradually improved 3 months post-treatment. A 75% improvement was recorded in 63% of the patients and 50% improvement in 91% of the patients 12 months post-therapy, indicating that autologous SVF for OA is safe and clinically effective [32].

## Regulatory Challenges

Translating cellular products from the laboratory to the bedside has been the goal of researchers, and a serious concern for clinicians and regulatory bodies [36]. In the USA, the Cell Therapy Regulatory Arm of the Food and Drug Administration (FDA) currently defines SVF as a drug, and/or biologic product based on the following standards: (1) non-homologous usage: under the Code of Federal Regulations (CFR) Title 21 (Human Tissue Intended for Transplantation), adipose tissue is classified as structural tissue that is intended to cushion and support other tissues. This means that, when SVF products are used for non-adipose-related conditions, the user must follow FDA drug regulatory standards. (2) Addition of enzymes: collagenase, an enzyme that breaks peptide bonds, and extracellular structures, puts SVF above minimally manipulated cells limit, according to Title 21 CFR 1271.10(a.1). The last one is (3) removal of structural components: during the digestion and washing process of SVF, the structural components of adipose tissue are removed, which falls outside of its natural biologic function [16, 25].

In Canada, autologous SVF administration meets the definition of “drug,” and persons who prepare (manufacture) and administer (distribute) them must comply with Sects. 8 and 11 of the Food and Drugs Act [37]. In addition, like other new drugs, SVF must be authorized by Health Canada, except when it is minimally manipulated, and is intended for homologous therapy. However, as investigational drug MAT-SVF are also subject to clinical trial requirements under Division 5 of the Food and Drug Regulations (FDR) [37].

In Europe, SVF and other cellular products are classified as advanced therapy medicinal products (ATMPs) and strictly regulated through the European Medicines Agency (EMA) [24]. However, the Directive 2004/23/EC and 1394/2007 of the EMA lowered the bar on autologous SVF treatment conducted in the same surgical procedure, as such that essential functions of cells as in the donor’s adipose tissue are preserved (https://www.ema.europa.eu/ema) [16].

In Australia, SVF is somewhat exempted from regulation. Therapeutic Good Administration (TGA), which enforce the regulation of human cell and tissue products, excluded SVF, cells, and tissue products from regulation, provided they are collected from a patient, under the clinical care of a licensed medical provider, and these cellular product manufactured by the same medical provider for the therapeutic application in the treatment of a single indication and in a single course of treatment of that patient, or by persons under the professional supervision of the same medical provider (https://www.tga.gov.au) [16, 35].

In most Asian countries, SVF is considered a low-risk therapy. The Japanese Act on the Safety of Regenerative Medicine guidelines [20] classified SVF as low risk or medium risk, depending upon the level of risk associated with the medical treatment. However, even as a low-risk category, the regenerative medicine plan, must be submitted to the Ministry of Health, Labor and Welfare, and treatment providers must report any adverse effect and specific plan details [16, 36].

The US FDA, Canadian FDR, and European EMA have similar regulations that allow the distribution of cell therapy products in their respective jurisdictions; however, slight differences exist in contents and structure. The European Union has regulations specifically for cell therapy products, while the US FDA and Canada FDR apply existing drug regulatory frameworks [16, 25]. In comparison, the USA holds the most stringent regulations for SVF, as the final SVF/MAT product still possesses unanswered clinical safety concerns [37].

## Limitations

SVF treatment can cause side effects such as swelling and tendonitis, which may limit its applicability in some patients [15]. Several production protocols have been developed; however, there is no gold standard protocol, thereby creating variations in the final product [11, 14, 29]. Variabilities also exist in therapeutic dosages and outcomes. Most reports on SVF have been autologous, which possesses minimal immune risks; however, the availability of healthy autologous therapy might be scarce in some situations, posing morbidity challenges to patients [6]. Allogeneic therapies may be an ideal off-the-shelf treatment that could limit autologous production time, and enable a selection of more healthy donors [10]; however, there are concerns regarding long-term immunogenicity [28]. While isolated adult mesenchymal cells have shown initial immune privilege in allogenic treatments, SVF has not and may pose serious immune challenges [10]. Variabilities in constituent cell counts may also be challenging [24, 33]. Hence, SVF cannot currently be produced in standard doses, strength, and purity owing to varieties in cell proportions of the final cell isolate. Other issues include understanding the mechanism of action and how long the cells stay in the joint to exert their effects. It is also not known if SVF obtained from different adipose depots of the body would yield the same result. Lastly, the majority of studies on SVF for knee regeneration are preclinical investigations and case reports. More randomized clinical studies are required to understand SVFs positive impacts on OA.

## Summary and Conclusion

Cartilage degeneration in OA is multifactorial [4]; however, inflammatory mechanisms have been implicated as chief agents for structural degeneration and osteophyte formations [1]. Chondrocytes maintain ECM function and regulate cartilage homeostasis. Aging factors, including inflammation and oxidative stress, disrupt homeostasis predisposing cartilage to degradation [16], and adenosine deficiency-mediated apoptosis, due to decreased mitochondrial electron transport chain [20]. Thus, the reduction in adenosine triphosphate (ATP) leads to decreased adenosine in the ECM, which promotes OA inflammatory changes [20]. This inflammatory theory has increased interest in the anti-inflammatory potentials of cellular products for more effective non-operative OA treatment modalities that may prevent disease progression and heal cartilage that has degenerated [3].

**Fig. 3:**
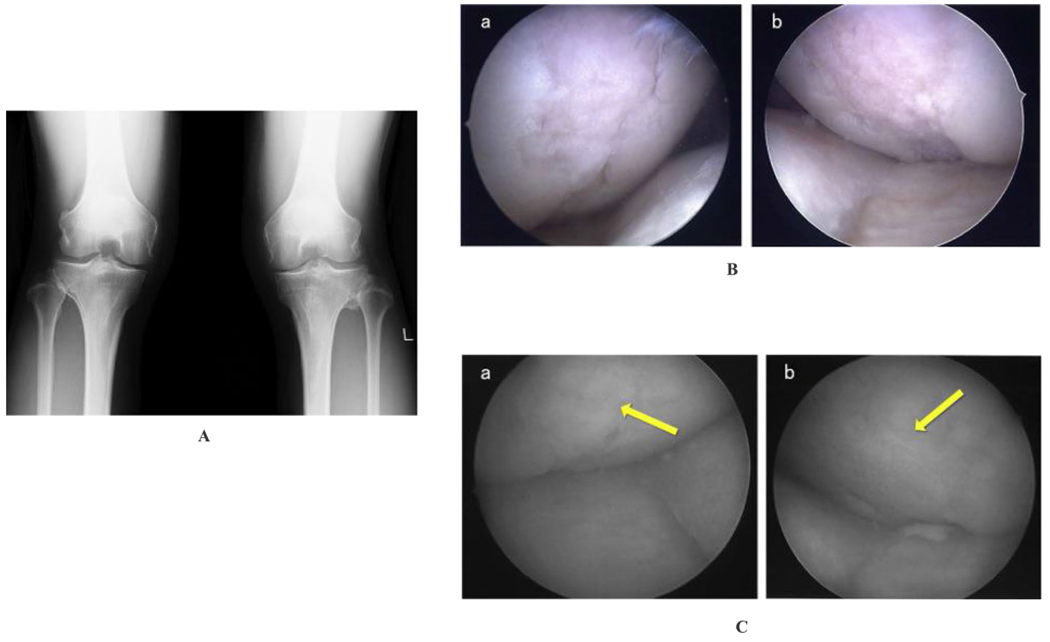
**A** X-ray was taken in a weighted postero-anterior view of the knee at 45° flexion (Rosenberg’s view), showing a slight and moderate medial joint space narrowing in the right and left knee, respectively (KL grades II, III). **B** Preoperative arthroscopic findings in the right and left medial femoral condyles show 2.5 × 1.5 cm cartilage defect in the right knee (a), and 3 × 1.5 cm cartilage defect in the left knee (b). **C** Second-look arthroscopic findings in the right and left medial femoral condyles 6 months post-operation revealed that the cartilage defect area was covered by regenerated cartilage. ((a) right), ((b) left). Adapted from Yuma Onoi et al., 2019 [8]

The clinical applications of adipose-derived cellular products, including MAT-SVF, are making way to the clinics globally [14, 20, 29, 35]. On the other hand, SVF and ADSCs have also shown great therapeutic potential [25]; however, due to their relatively non-minimal manipulations, and the regulatory challenges involved, they have limited use in the clinic. With published evidence of SVF’s efficacy in OA cartilage regeneration (Fig. 3) [8, 25], many patients currently seek this investigational therapy. With more widespread acceptance, there may be a greater tendency its use at an earlier phase of cartilage degeneration/OA. This may in fact result in improvement in results [6, 10].

**Fig. 4:**
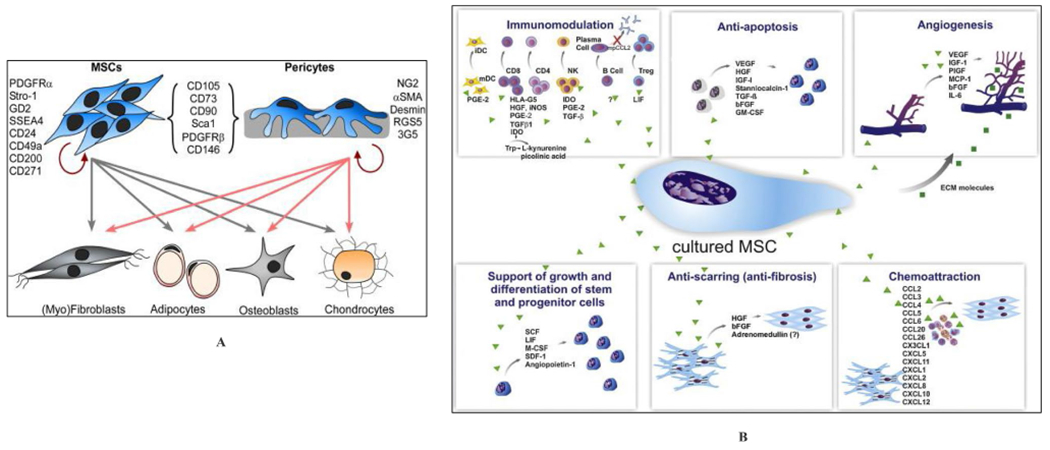
**A** Schematic diagram illustrating the capacity for MSC and pericyte differentiation into separate lineages (gray and pink arrows) and self-renewal (red arrows). The unique and shared markers of both cell types are shown. Adapted from Suet-Ping Wong et al., 2015, Ref (#^39^). **B** Paracrine effects of MSCs. The secretion of a broad range of bioactive molecules is believed to be the main mechanism by which MSCs achieve their therapeutic effect and it can be divided into six main categories: immunomodulation, anti-apoptosis, angiogenesis, support of the growth and differentiation of local stem and progenitor cells, anti-scarring, and chemo-attraction. Although the number of molecules known to mediate the paracrine action of MSCs increases every day. Adapted from Meirelles et al., 2009, [38]

However, before either MAT-SVF or SVF achieves widespread clinical availability, more studies need to focus on manufacturing validation, and proof of pharmacological activity [15, 21]. The mechanism of action of MAT-SVF is not also fully understood (Fig. 4) [38, 39]; however, current data indicates that it can improve pain and restore joint function by (1) direct adherence and integration to degenerative host tissue for growth and differentiation and/or (2) trophic effects resulting from the secretome of constituent cells in SVF [16, 24].

On direct engraftment, constituent cells are attracted by degenerative and inflammatory stimuli. They migrate to the target tissues by surface molecules and chemokine receptors (CXCR4, integrins, selectins, vascular cell adhesion molecule-1, etc.) associated with cell migration [12, 15]. It has also been reported that ATP is released from damaged cells into the extracellular space, triggering immune signals and immune cells to migrate and accumulate at the damage site to remove dying cells [20]. Following the inflammatory stimulus, stem/progenitor cells further attach to and migrate across the endothelial cells to reach their target tissues. Thus, SVF cells, like leukocytes, bind to endothelial cells and migrate to the injury site, followed by adhesion on the ECM [19].

The trophic bioactive factors secreted by the SVF cells can be categorized into (1) growth factors/cytokines and (2) extracellular vesicles [20]. SVF produce a variety of anti-inflammatory growth factors and cytokines, including the hypoxia-inducible factors (HIF), basic fibroblastic growth factor (bFGF), tumor necrosis factor-alpha (TNF-α), transforming growth factor-β1 (TGFβ1), insulin-like growth factors (IGFs), vascular endothelial growth factor (VEGF), and interleukins, IL10, IL13, and IL18-binding protein (IL18BP); IL1 receptor antagonist (IL1RA); and anti-apoptotic proteins, etc. [20, 28]. They inhibit the production of pro-apoptotic factors, and thus, the final regenerative result is determined by the net effect of these cytokines interacting together [33].

Extracellular vesicles (EV) are membrane vesicles that are released by a variety of cells into the extracellular space, that can be divided into apoptotic bodies, exosomes, and microvesicles [20]. When released from stem/progenitor cells, they contribute to the regeneration of cartilage via paracrine-like actions [4]. They transfer bioactive cytoplasmic components such as nucleic acids, mitochondria adenosine, lipids, and proteins from SVF constituent cells to recipient cells in the degenerating/inflammatory tissue [20]. For the major biologics that facilitate the above mechanisms, a description of some of the constituent cells in SVF are given below:

Endothelial progenitor cells (EPCs) are required for vasculogenesis during early embryo development; however, in adults, vascular growth develops from fully differentiated endothelial cells through angiogenesis [29]. Thus, SVF, which contains EPCs and endothelial cells at percentages varying between 7 and 30%, provides a possible rationale for its use in the treatment of diseases associated with inflammations [16].

Hematopoietic stem cells (HSC) are cells isolated from the blood/bone marrow that possess self-renewal properties and plasticity, able to differentiate to a variety of specialized cells within the mesodermal origin. Hence, the presence of HSCs in the SVF suggests that SVF could reliably promote tissue regeneration [21, 33].

Immune cells, such as monocytes and macrophages, make up about 10% of the total SVF cells, based on CD14 expression [16]. The macrophages are further divided into M1 and M2 macrophages [19]. M1 produce pro-inflammatory factors, which inhibits the chondrogenic differentiation of MSCs, while M2 expressing CD163 and integrin avb5, supports the survival of chondrocytes by producing anti-inflammatory cytokines (TGF-β1, IL10, and IL-1 receptor antagonist), thus attenuating the deteriorating effects of inflammatory cytokines such as TNF-α and IL-1[16, 20].

Pericytes are one of the main cells of the blood vessels in which their recruitment is essential for vasculature maturation [6, 20]. They perform different functions in different organs, but like smooth muscle cells, pericytes regulate blood flow on large vessels by modulating vasoconstriction and vasodilation [28]. When the growth of a newly formed vessel in adipose tissue ceases, they secrete growth factors such as platelet-derived growth factor-beta (PDGF-B) that attract pericytes to envelop the vessels [18]. The density of pericytes in adipose tissue ranges between 10 and 70%, and studies have shown that SVF-derived pericytes containing (CD146, CD452, CD342, and CD312) have significant bone regeneration potential [16].

In conclusion, SVF and MAT-SVF have provided a better quality of life to patients, with confirmatory evidence from images of regenerated joints. FDA currently has approved MAT-SVF obtained and administered at the point of care.

## Future Directions

The significant clinical burden of knee OA in the population highlights the importance of finding less invasive regenerative strategies [40–44]. Regenerative engineering offers the prospect for new treatments and new paradigms for achieving healing and tissue regeneration [45–47]. For SVF treatment, patients may need repeated injections to experience the full effects of treatment on cartilage, and thus, future directions may include provisions for product storage and banking for future autologous therapies at the time of initial liposuction/treatment.

It would be beneficial to the medical community if the underlying mechanisms of SVF could be better understood [48–52]. A specific focus of needed work is the examination of trophic versus constitutive effects to determine if SVF releases growth factors and cytokines to stimulate cartilage regeneration, or whether the cells of the SVF build up cartilage themselves [53–58]. If the mechanism points towards cytokine release profile rather than the actual regeneration of cartilage from the components cells of SVF [26, 59], it would encourage research towards the isolation and production of these implicated factors, thus saving patients from surgical tissue harvest, and costly procedures such as liposuction [51, 59–63]. Future studies comparing the secretome composition and regenerative effect of mechanically versus enzymatically processed SVF may elucidate part of the underlying mechanism of action of SVF as well as the importance of the cell compositions [26, 58, 64].

Lastly, with advancements towards 3-dimensional cell printing [55, 64–66], it would be interesting to evaluate the effect of printing the SVF-like extracellular matrix in a hydrogel scaffold for osteochondral defects, or incorporating SVF into an injectable hydrogel for enhanced retention and viability in the local environment as has been demonstrated in previous studies using adipose-derived stem cells [67].

## Figures and Tables

**Table 1:** A list of different cell therapy companies that have developed new processing kits/technologies for the SVF and MAT-SVF products

Company	Device/product	System	Operation	Isolation method	Manipulation Class	Suitable Class
IntelliCell Biosciences Inc	cGTP cellular lab	Semi-closed	Automated	Agitation/centrifugation	Minimal	SVF
Cytori Therapeutics Inc	Celution 800/CRS	Semi-closed	Automated	Decantation	Major-Celase™ used	SVF
GE Healthcare (Biosafe Gp SA)	Sepax	Semi-closed	Semi-Automated	N/A	Minimal	MAT-SVF
Tissue Genesis Inc	Tissue Genesis Icellator	Semi-closed	Semi-Automated	N/A	Major adipase used	SVF
Cellular Biomedicines, Inc., China	Re-Join® Therapy	Semi-closed	Semi-Automated	N/A	Major	SVF
Cellular Biomedicines, Inc., China	AlloJoin® Therapy	Semi-closed	Semi-Automated	N/A	Major	SVF
Human med AG INC	ADIPOA-2	Closed	Automated	Decantation	Minimal	MAT-SVF
InGeneron, Inc	Transpose® RT System	Closed	Automated	Centrifugation, Agitation	Major	SVF
Lifecell CO (Allergen PLC)	Alloderm®	Closed	Automated	N/A	Minimal	MAT-SVF
PNC International Co., Ltd	Cha-Station™	Semi-closed	Semi-Automated	N/A	Minimal	MAT-SVF
PNC International Co., Ltd	Multi-Station	Open	Manual	Centrifugation	Minimal	MAT-SVF
GID Group, Inc	GID SVF-1	Closed	Manual	Filtration	Major GIDzyme used	SVF
Medi-Khan	Lipokit with Maxstem	Semi-closed	Manual	Centrifugation	Minimal	MAT-SVF
Eurosilicone	Puregraft 250	Semi-closed	Manual	Filtration	Minimal	MAT-SVF
Proteal®	Stem.pras with Duografter II®	Closed	Manual	Decantation	Minimal	MAT-SVF
Tulip medical product	Tulip Fat Transfer	Closed	Semi-automated	Decantation	Minimal	MAT-SVF
Cytori Therapeutics	Celution® System	Closed	Automated	Disaggregation	Minimal	SVF
Cellthera, s.r.o., Brno	Cellthera Kit I	Closed	Semi-automated	Disaggregation	Major	SVF
Cellthera, s.r.o., Brno	Cellthera Kit II	Closed	Automated	Disaggregation	Minimal	MAT-SVF
(Lipogems International Spa, Milan, Italy)	Lipogems® processing kit	Closed	Automated	Decantation	Minimal	MAT-SVF
